# Colchicine in Managing Skin Conditions: A Systematic Review

**DOI:** 10.3390/pharmaceutics14020294

**Published:** 2022-01-27

**Authors:** Stefano Dastoli, Steven Paul Nisticò, Pietro Morrone, Cataldo Patruno, Antonio Leo, Rita Citraro, Luca Gallelli, Emilio Russo, Giovambattista De Sarro, Luigi Bennardo

**Affiliations:** 1 Department of Health Sciences, Magna Graecia University, 88100 Catanzaro, Italy; stefanodastoli@gmail.com (S.D.); steven.nistico@gmail.com (S.P.N.); cataldopatruno@libero.it (C.P.); aleo@unicz.it (A.L.); citraro@unicz.it (R.C.); gallelli@unicz.it (L.G.); erusso@unicz.it (E.R.); desarro@unicz.it (G.D.S.); 2 Azienda Ospedaliera di Cosenza, 87100 Cosenza, Italy; morronepietro1973@libero.it

**Keywords:** colchicine, dermatology, pustular dermatoses

## Abstract

(1) Background: Colchicine is a natural alkaloid with anti-inflammatory properties used to treat various disorders, including some skin diseases. This paper aims to incorporate all the available studies proposing colchicine as a treatment alternative in the management of cutaneous conditions. (2) Methods: In this systematic review, the available articles present in various databases (PubMed, Scopus-Embase, and Web of Science), proposing colchicine as a treatment for cutaneous pathological conditions, have been selected. Exclusion criteria included a non-English language and non-human studies. (3) Results: Ninety-six studies were included. Most of them were case reports and case series studies describing colchicine as single therapy, or in combination with other drugs. Hidradenitis suppurativa, pyoderma gangrenosum, erythema nodosum, erythema induratum, storage diseases, perforating dermatosis, bullous diseases, psoriasis, vasculitis, acne, urticaria, stomatitis, actinic keratosis, and pustular dermatosis were the main diseases discussed in literature. Although the therapeutic outcomes were variable, most of the studies reported, on average, good clinical results (4) Conclusions: Colchicine could be, as a single therapy or in combination with other drugs, a possible treatment to manage several skin diseases.

## 1. Introduction

Colchicine is a natural alkaloid derived from the plant *Colchicum autumnale*. Nowadays, it is used in various conditions, including gout, familial Mediterranean fever, and in different rheumatological conditions. It is administered orally, at a dosage varying from 0.5 to 2 mg per day. Its metabolism is primarily and quickly hepatic; after that, the drug is excreted through the bile and the kidneys [1]. Plasmatic concentrations and bioavailability may vary between individuals, so the profile of the response and adverse events (AEs) to colchicine may vary accordingly. The AEs are mainly gastrointestinal symptoms, such as vomiting, diarrhea, and nausea, and affect up to 10% of patients treated with the labelled dosage. Other AEs include muscular damage and anagen or telogen effluvium, according to the drug dosage [2,3,4]. Other AEs, such as cardiac arrhythmias, cardiac failure, kidney disease, and myelosuppression are related to the overdosage.

Colchicine accumulates at a higher dosage in leukocytes than in other blood cells, reducing their functional ability, especially in neutrophils, as well as reducing their migration and their ability to degranulate by inhibiting microtubule polymerization. This accumulation reaches its peak about 48 h after drug assumption. Colchicine also seems involved in inhibiting the cyclooxygenases COX-1 and COX-2. There is no clear limit that establishes the therapeutic, toxic, and lethal doses of colchicine in children or adults.

The lowest reported lethal dose of oral colchicine is 7 mg. In contrast, adult patients survived acute ingestions of dosages up to 60 mg. The oral LD50 of colchicine in mice is 5.87 mg/kg, with a pKa of 1.85 [5].

Given this anti-inflammatory effect, this drug has been initially proposed for the management of various inflammatory and neutrophilic skin diseases, such as pustular dermatosis [6].

**Figure 1:**
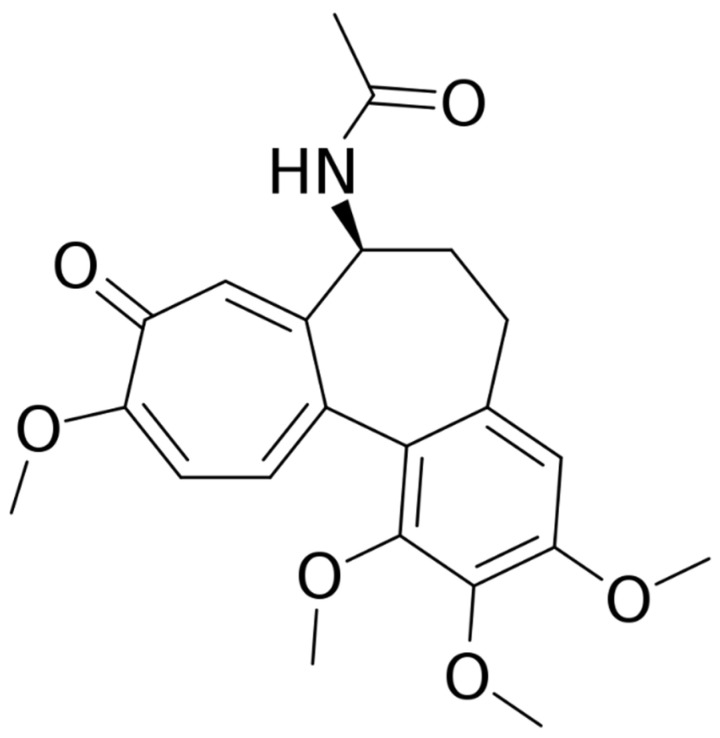
Structure of colchicine.

Other uses of the drug in dermatology have been proposed during the last decade. This systematic review of the international literature aims to explore the state-of-art use of colchicine in dermatology, including all available studies, case series studies, and reports to guide clinicians in the correct drug choice in uncommon clinical conditions. The structure of colchicine is reported in Figure 1.

## 2. Materials and Methods

The authors followed the criteria established in the preferred reporting items for systematic reviews and meta-analyses (PRISMA) guidelines for this review [7].

### 2.1. PICO Question

In what dermatological conditions is the use of topical or oral colchicine an effective treatment?

### 2.2. Search Strategy

A comprehensive literature search to identify relevant studies was carried out by two researchers (L.B. and S.D.) from the 1st of July 2021 up to the 28th of November 2021, with no temporal restriction, using the following databases: MEDLINE/PubMed (National Center for Biotechnology Information, NCBI), EMBASE(Ovid), Google Scholar, and the Cochrane Central Register of Controlled Trials (CENTRAL). The search string contained medical subject headings (MeSH) and free-text terms. The keywords used to select the maximum number of pertinent studies included: “colchicine” AND “skin”, “psoriasis”, “urticaria”, “vasculitis”, “pyoderma gangrenosum”, “palmoplantar pustulosis”, “Sweet Syndrome”, ”pustular dermatosis”, “pemphigus”, “pemphigoid”, “dermatitis herpetiformis”, ”epidermolysis bullosa”, ”acne”, “hidradenitis”, ”stomatitis”, and “actinic keratosis”. In addition, a search for citations in the reference lists of the selected articles and reviews was conducted in order to identify potentially missing studies.

### 2.3. Inclusion and Exclusion Criteria

The (PRISMA) methodology selected studies based on the following criteria. Only studies that met the following inclusion criteria were included: (a) Papers in the English language and (b) all cited studies must have been approved by an ethics committee or an institutional review committee. Exclusion criteria were: (a) papers that are not relevant to the PICO question and (b) studies that do not provide sufficient data on the topic or when the paper was not available. Observational studies, case series studies, and case reports were included. After eliminating the duplicates, the eligible articles were screened based on the title and abstract; finally, the full text of the articles that were potentially suitable for their inclusion in the review was analyzed. In case of discrepancies between the researchers, a third physician (C.P.) had the final word on the inclusion or exclusion of the paper. In addition, the studies were rated using a modified Oxford Centre for Evidence-Based Medicine 2011, and the levels of evidence were assigned to each association [8] (see Table 1). The assigned levels of evidence were discussed among members until a consensus was reached.

## 3. Results

**Figure 2:**
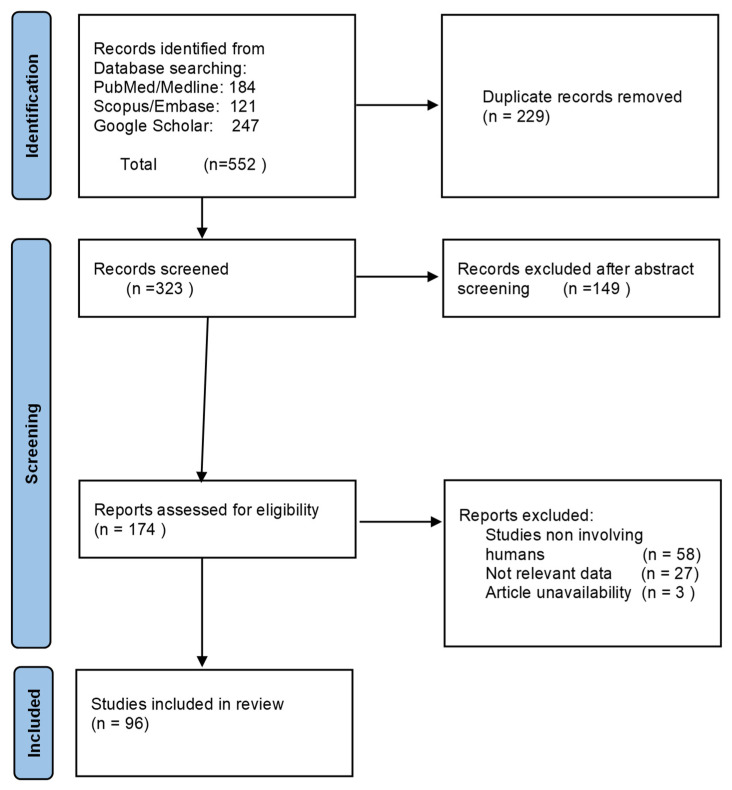
PRISMA flow chart of study selection.

The search identified 552 references. The screening of titles and abstracts removed 378 references. The full text of the remaining 174 papers was assessed for inclusion, and finally, 96 papers met the inclusion criteria and were included in the review. The article selection flow chart [9] (see Figure 2) summarizes the search strategy adopted in this study. Oral colchicine has been proposed in various dermatological conditions, with different levels of evidence and results (Table 2). The drug dosage varies from 0.5 to 2.4 mg per day. Most articles were case reports or small case series studies, lacking clinical trials.

This was especially true for some skin diseases, namely, neutrophilic eccrine hidradenitis, plantar eccrine hidradenitis, pyoderma gangrenosum, erythema nodosum, erythema induratum, cutaneous amyloidosis, cutaneous sarcoidosis, hereditary angioedema, acquired perforating dermatosis, linear IgA bullous disease, pemphigus, pemphigoid, epidermolysis bullosa, dermatitis herpetiformis, and subcorneal pustular dermatosis. Further studies and/or clinical trials are, therefore, needed in order to confirm the results reported in this review. Other conditions, such as psoriasis, palmoplantar pustulosis, leukocytoclastic vasculitis, hidradenitis suppurativa, acne, and urticaria reported contrasting results in clinical trials. Therefore, colchicine’s effectiveness in managing these conditions has not definitely been proven.

For other conditions, such as various types of stomatitis, some subtypes of vasculitis including Behçet’s disease, and the topical management of actinic keratosis, colchicine has robust evidence and could be considered as a first-line treatment option. The rate of AEs observed in the reported studies was considered low, especially when the dosage did not exceed 1.5 mg of colchicine per day.

## 4. Discussion

### 4.1. Psoriasis

**Figure 3:**
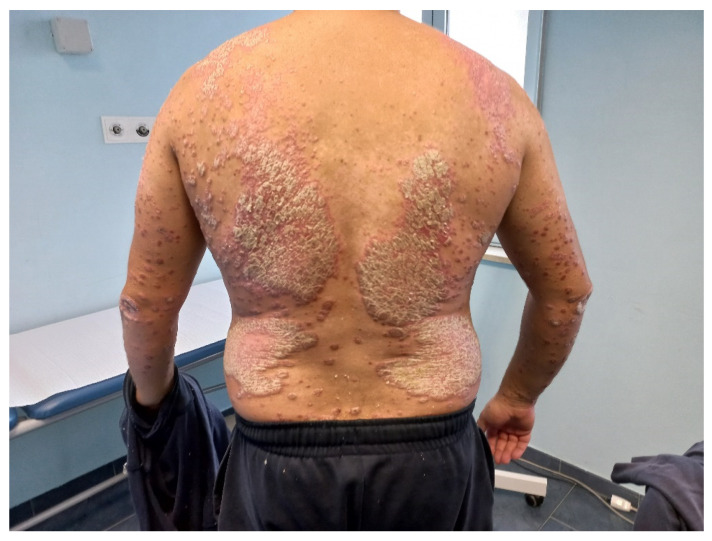
Plaque psoriasis (courtesy of Dr. Bennardo).

Psoriasis is characterized by erythematous-squamous plaques affecting mainly extensor surfaces but spreading to all body areas (Figure 3). Different clinical phenotypes of psoriasis have been reported, including palmoplantar, inverse, guttate, pustular, and others. It may be associated with comorbidities, such as arthritis [10,11,12]. The currently approved therapies include topical corticosteroids and vitamin D derivatives [13,14,15], as well as systemic therapies based on traditional immunosuppressive drugs, such as methotrexate and cyclosporine, and novel biologic drugs targeting tumor necrosis factor (TNF) alpha, anti-interleukin (IL) 17, and anti-IL 23 [16,17,18,19,20]. Topical or systemic colchicine was proposed in the late 1970s and early 1980s to manage this condition [21,22].

Kadbey et al. reported the improvement of plaque psoriasis with topical 1% colchicine in 12 subjects [22]. Wahba and colleagues treated 22 patients at a dosage of 0.02 mg per kg per day. Eight out of nine patients that reported mild psoriasis showed a complete or an almost complete clearing, and patients affected by arthritis reported a reduction of joint pain. Patients suffering from more severe forms of psoriasis reported minimal-to-no clearing. The authors administered colchicine to five patients previously treated with systemic drugs, such as methotrexate, and reached skin clearance. These subjects maintained the results for 8 months in approximately 80% of the cases [23]. A Japanese group described the case of a 32-year-old woman responding to a combination therapy of methotrexate and colchicine [24]. Zachariae and colleagues reported a complete remission of the disease in three-quarters of their patients [25]. In a double-blinded, placebo-controlled crossover study on patients with psoriatic arthritis, the drug was efficacious on the arthropathic component, but not on the skin lesions [26]. On the other hand, a Canadian two-center, double-blind, crossover study on colchicine did not prove superior to a placebo in managing psoriatic arthritis [27]. Stefanaki and colleagues described a patient affected by palmoplantar psoriasis and subcorneal pustular dermatosis previously treated with dapsone with no response. Therapy with colchicine 1.5 mg/day, then lowered to 0.5 mg per day, led to the complete resolution of both conditions at the 12-month follow-up [28].

Recently, a Japanese case report described two patients treated with anti-IL monoclonal antibodies (one patient with anti-IL17 secukinumab, and the other with anti-IL23 guselkumab) with no control of the skin disease. This reached an excellent clinical response after the addition of 1.5mg of colchicine per day without any AEs [29].

Kagan and colleagues reported, in a patient with amyloidosis and psoriatic arthritis, a good efficacy of colchicine on arthritis, as well as the reversal of nephrotic syndrome associated with amyloidosis [30]. The evidence for using colchicine for this condition is contrasting, but this drug could be proposed in pustular or arthropathic variants that do not respond to conventional therapies.

### 4.2. Palmoplantar Pustolosis

Palmoplantar pustulosis is an uncommon, chronic, pustular condition characterized by the presence of sterile pustules on the palms and soles [31]. Acrodermatitis continua of Hallopeau may be considered a subvariant, affecting single fingers [32]. Colchicine has been traditionally used to manage this subvariant, with contrasting results. Takigawa and colleagues treated 32 patients by using a dosage that varied from 1 to 2 mg per day, decreasing the dose to 0.5/1 mg per day in responding patients. Thirteen patients showed the complete disappearance of pustules, while 14 patients showed a reduction in pustules. One patient did not benefit from the treatment, and four stopped the treatment due to its side effects, mainly nausea and diarrhea. Eight cases experienced a reactivation of the condition 3 months after the treatment discontinuation [33]. Twenty-two patients affected by palmoplantar pustulosis entered a double-blind, crossover trial, using oral colchicine at the dosage of 0.5 mg twice per day or a placebo. No significant improvement on the skin condition was recorded [34]. English and colleagues treated 10 patients with no significant difference compared to the placebo [35]. A Danish group performed a double-blind crossover trial on 27 patients treated with 0.5 mg of colchicine three times daily; 10 patients only experienced a reduction in pustule formation, whereas the redness, scaling, and subjective symptoms were unchanged, and a high incidence of gastrointestinal side effects was reported [36]. Wong and colleagues reported a case series stduy of three patients affected by severe palmoplantar pustulosis and their significant improvement of the condition after the treatment with colchicine [37]. Adisen et al. retrospectively analyzed 52 patients, and 25 of them were treated with colchicine. In 15 out of 25, a substantial improvement was observed, with one partial responder and nine non-responders [38].

### 4.3. Neutrophilic Eccrine Hidradenitis

Neutrophilic eccrine hidradenitis is a very uncommon neutrophilic condition that is histologically characterized by the necrosis of the eccrine glands, as well as a local neutrophilic infiltration. It is usually observed in patients affected by acute leukemia or other malignancies during chemotherapy. Belot and colleagues described an idiopathic case of a middle-aged female patient treated with colchicine, reporting clinical improvements after just one month [39].

### 4.4. Eccrine Hidradenitis

Eccrine hidradenitis, or idiopathic hidradenitis, is an uncommon condition affecting children and young adults characterized by tender, red lumps on the soles of the feet, and less often, on the palms. Histologically, a neutrophilic and lymphocytic infiltration is present at the base of the eccrine glands. Scheer and colleagues reported the efficacy of a 5-month cycle of 0.5 mg per day of colchicine in a 7-year-old boy, with no AEs. The condition disappeared after 3 days, but colchicine was continued as a preventive measure [40].

### 4.5. Hidradenitis Suppurativa

**Figure 4:**
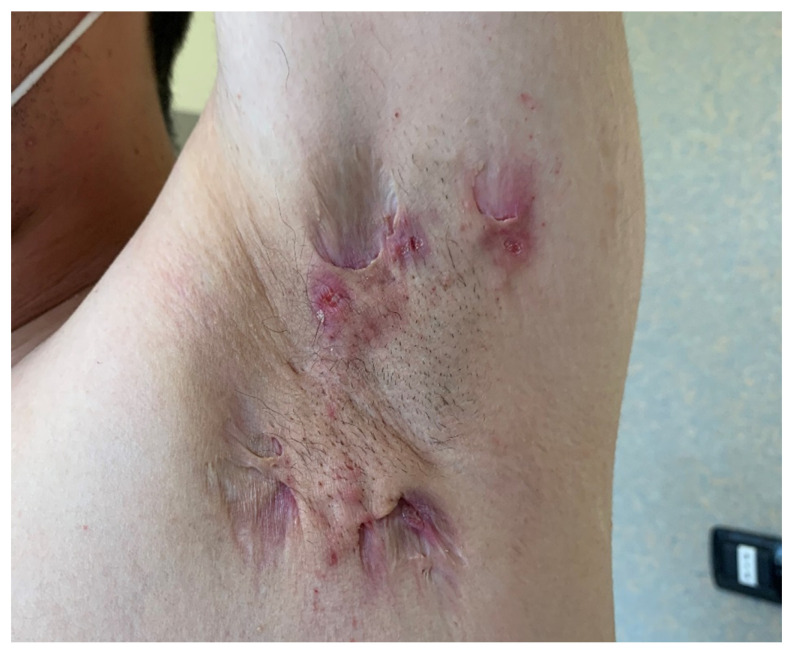
Hidradenitis Suppurativa (courtesy of Dr. Dastoli).

Hidradenitis suppurativa (HS) is a chronic inflammatory skin disease affecting areas with a high density of apocrine glands and is characterized by subcutaneous nodules that may evolve into fistulas with purulent secretion (Figure 4) [41,42,43]. Various treatments have been proposed to manage this condition, such as topical and systemic antibiotics, as well as retinoids [44], with no lasting results [45]. Recently, biologic drugs, such as anti-TNF alpha and anti-IL agents traditionally used to manage other chronic inflammatory diseases [46] have been proposed, with inconsistent results. HS is often associate with a high impairment in the quality of life of patients [47,48,49].

An open, prospective pilot study evaluated the efficacy of colchicine at 0.5 mg twice per day for up to 4 months in the management of HS. Colchicine treatment did not result in a clinically relevant improvement of disease severity. Some patients experienced nausea and diarrhea as AEs [50].

An open prospective study treated 20 patients (10 females and 10 males) with a combination of 100 mg of minocycline administered orally once per day and 0.5 mg of colchicine administered twice per day, for 6 months, followed by a maintenance regimen of 0.5 mg of colchicine twice a day for 3 months. The efficacy of the treatment was evaluated every 3 months, for 9 months, using the Physician’s Global Assessment scale, the Hurley scoring system, and the Dermatology Life Quality Index. All the patients showed an improvement in all the scores after 3 months of treatment and continued to improve over time, suggesting colchicine as a good agent in combination therapy [51]. A Greek study divided 44 patients into three groups, where the first group received 1 mg per day of colchicine as monotherapy, the second group received both colchicine and doxycycline at 100 mg per day, and the third group received both colchicine and doxycycline at 40 mg per day. The three groups reported an improvement in all measured scores, but there was no statistically significant difference among the groups [52].

These data seem to suggest that colchicine may be useful in treating HS, especially when associated with antibacterial drugs.

### 4.6. Acne Vulgaris

Acne is an inflammatory condition characterized by the blockage and inflammation of the pilosebaceous unit, leading to papulopustular lesions of the affected areas. The regions that are more commonly involved are the face, the back, and the chest. It usually presents during adolescence, but it may appear at any age. It is crucial to treat acne as soon as possible, as the more severe forms may lead to scarring [53,54,55]. Current standard treatments include topical and systemic antibiotics, topical and systemic retinoids, and keratolytic agents [56,57]. Photodynamic therapy has also been proposed [58]. An Italian study reported 12 patients with severe acne vulgaris that were treated for 2 months with colchicine at 1 mg per day, showing no improvement [59]. A Korean group described a 31-year-old female treated for acne conglobata with a combination therapy of isotretinoin, colchicine, and cyclosporin, with reasonable control over the condition [60]. An Iranian group treated 22 patients with severe acne that was resistant to conventional therapies with colchicine at 1 mg per day for 2 months. All patients improved up to 70%, with better results observed in cystic nodular acne with severe inflammation. No severe side effects were observed [61]. Therefore, colchicine may be considered a third-line treatment of severe acne after retinoid’s lack of response.

### 4.7. Urticaria

Urticaria is a characterized by wheals and/or angioedema; it is defined as acute if it occurs for less than 6 weeks, or chronic (chronic urticaria, CU) if it lasts for longer than 6 weeks. Among CU, chronic inducible urticaria (CIU) and chronic spontaneous urticaria (CSU) are identified. The treatment of CU is challenging and currently includes second-generation antihistamines, omalizumab, and cyclosporine [62,63]. Colchicine has also been proposed to manage the disease. Thirteen patients affected by delayed pressure urticaria were enrolled in a double-blind placebo-controlled trial. The researchers were unable to demonstrate any effect of colchicine in managing the disease [64]. Another study retrospectively enrolled 36 patients affected by chronic urticaria treated with colchicine, reporting, in 15 patients, a complete response, and in five patients, a partial response. Three patients had to stop colchicine due to gastrointestinal symptoms, and four patients experienced recurrence after treatment discontinuation [65]. Nabazivadeh et al., in a case-control study, proposed colchicine in the management of 55 patients but did not show statistically significant improvements among subgroups, although colchicine was helpful in the management of the signs and symptoms for patients [66]. A retrospective cohort study on 221 patients affected by CU showed that patients treated with a multidrug therapy, including colchicine, had better control of symptoms [67]. The use of colchicine in the management of urticaria remains controversial.

### 4.8. Pyoderma Gangrenosum

Pyoderma gangrenosum (PG) is a neutrophilic dermatosis characterized by a painful, rapidly enlarging ulcer that may mimic various pathologic conditions. It may be associated with some systemic diseases, such as inflammatory bowel diseases and rheumatoid arthritis [68,69]. Different case reports describe the use of colchicine in PG. Lugassy and Ronnen described a case series study of three patients suffering from familial Mediterranean fever, where PG positively responded to colchicine [70]. Two cases of PG associated with inflammatory bowel disease that were refractory to other treatments were managed with colchicine at 1 mg per day with the resolution of all lesions in 3 months, suggesting colchicine as a first-line treatment for refractory PG [71]. A Greek group reported two patients affected by PG that were unresponsive to traditional therapies, who were treated with colchicine (in one case, as a single agent at 2 mg per day, and in the other case, in combination with systemic steroids), leading to complete healing [72]. Parren and colleagues described a patient affected by PG of the penis. The patient was treated with 0.6 mg of colchicine three times per day, with the complete disappearance of the lesion in 4 months [73]. The results of colchicine in treating PG are encouraging. However, further prospective studies will be necessary to confirm the results obtained by current reports.

### 4.9. Sweet Syndrome

Acute febrile neutrophilic dermatosis, also known as Sweet syndrome (SS), is a neutrophilic dermatosis characterized by skin, and sometimes mucosal, inflammation with blisters and fever. It may be associated with infections, inflammatory bowel disease, internal malignancies, rheumatological conditions, immunodeficiency, and various drugs. In case of treatment inefficacy, various immunosuppressants have been experimented with [74,75]. A Japanese group described a 29-year-old woman affected by SS responding to 0.5 mg of colchicine three times a day after failing previous treatments with potassium iodide and oral prednisone (10 mg per day). The patient started to respond 2 days after the beginning of therapy, with a reduction of plaques and aphthae. A complete response was obtained in 1 week [76]. The same group successively reported a case series study of three patients affected by SS that were treated with 1.5 mg of colchicine daily, reporting the condition’s resolution in 2–5 days [77]. Twenty patients with SS, treated with colchicine at 1.5 mg or 1 mg per day, were retrospectively enrolled by a single institution. Eighteen of them responded to therapy, reporting the disappearance of fever, arthralgia, and cutaneous lesions in 2–5 days. Only one patient did not tolerate the treatment, with diarrhea and vomiting, while two patients did not have any improvement [78]. Ritter and colleagues described the case of a woman affected by SS that was unresponsive to corticosteroid treatment. The use of colchicine 0.6 mg twice daily led to the resolution of SS but it had to be continued for almost 10 years to avoid new cutaneous relapses [79]. A Thai group reported the case of a patient affected by a bullous SS responding to a combination therapy of acitretin and colchicine [80]. Colchicine remains a mainstay in the management of SS in patients that are not responsive to systemic corticosteroids.

### 4.10. Erythema Nodosum

Erythema nodosum is an inflammatory condition involving the subcutaneous fat (panniculitis). It may be associated with inflammatory intestinal conditions, such as Crohn’s disease [81], bacterial and viral infections, as well as drugs and vaccines [82]. Systemic steroids, non-steroidal anti-inflammatory drugs, and oral potassium iodide may be used to manage this condition. Colchicine has been reported as a possible treatment, although no clinical study is present in medical literature, only old case reports [83].

### 4.11. Actinic Keratosis

Actinic keratoses (AK) are precancerous lesions found on photodamaged skin [84]. They may be considered a precursor of squamous cell carcinoma, characterized by the abnormal and quick growth of keratinocytes in the epidermis, often secondary to chronic ultraviolet or sunlight exposure [85,86]. Various treatments have been traditionally proposed to manage this condition, such as topical imiquimod, sodium-diclofenac, piroxicam, 5-fluorouracil, cryotherapy, photodynamic therapy, and surgery [87,88]. Topical colchicine was proposed to manage such conditions since the late 1960s [89]. A Swiss group recruited 20 patients with hypertrophic, multiple AK on the forehead. Ten of them applied 1% colchicine in hydrophilic gel twice daily, while the others were treated with the vehicle. Seventy percent of patients in the colchicine arm showed a complete response with no relapse at the 2-month follow-up; no systemic absorption was reported [90]. Another study tried to establish the correct concentration of topical colchicine, treating eight patients with a 0.5% colchicine cream and the other eight with a 1% colchicine cream for ten days. Both groups showed an overall improvement, with no statistically significant differences between the groups (a 73.9% reduction rate in the 1% group and a 77.7% reduction in the 0.5% group), showing equal efficacy for the 0.5% and 1% formulations [91]. A double-blind, randomized study compared the effectiveness of 1% colchicine cream and 3% diclofenac sodium gel to treat AK. Seventy patients were randomly allocated two one of the two groups. The results were similar in both groups, while the diclofenac-treated patients experienced a higher rate of side effects (especially erythema) [92]. A randomized controlled trial compared the effectiveness of 0.5% colchicine cream and photodynamic therapy with methyl-aminolevulinate activated by 636 nm of light in treating AK of the forearms, with similar results and side effects in both groups [93,94]. Topical colchicine seems as effective as diclofenac sodium gel and photodynamic therapy and could be used as a treatment alternative in recalcitrant cases.

### 4.12. Cutaneous Vasculitis

Cutaneous vasculitis (CV) is a heterogeneous group of disorders characterized by inflamed capillaries and veins in the dermis. They can be classified as capillaritis, small vessel vasculitis, medium vessel vasculitis, or large vessel vasculitis [95,96]. CV are generally characterized by petechiae, palpable purpura, and infiltrated erythema, indicating dermal, superficial, small-vessel vasculitis, or, less commonly, by nodular erythema, deep ulcers, livedo racemosa, and digital gangrene, implicating deep dermal, or subcutaneous, muscular-vessel vasculitis [97,98,99]. The treatment is usually anti-inflammatories with steroids and immunosuppressants [100]. Colchicine has been proposed in the management of various disorders in this spectrum of conditions.

#### 4.12.1. Erythema Elevatum Diutinum

Erythema elevatum diutinum is a rare form of small-vessel necrotizing vasculitis, characterized by red or dark papules on the backs of the hands and other extensor surfaces [101,102]. Although the gold-standard treatment is dapsone, colchicine has also been proposed as a second-line treatment in non-responsive cases. Henriksson et al. reported the case of a patient that was unresponsive to dapsone. A small cycle of oral colchicine at 0.5 mg twice per day led to the resolution of the condition [103]. A Turkish group reported another case suffering from dapsone-induced pancytopenia responding to 0.5 mg of colchicine twice per day, highlighting how this drug should be preferred in case of hematological conditions [104].

#### 4.12.2. Leukocytoclastic Vasculitis

Leukocytoclastic vasculitis is the most common form of cutaneous vasculitis, infesting small vessels characterized, histopathologically, by the immune complex-mediated vasculitis of the dermal capillaries and venules [105]. Its proposed treatments include corticosteroids, dapsone, hydroxychloroquine, and rituximab [106,107]. Plotnick and colleagues reported a case of a middle-aged woman affected by a leukocytoclastic vasculitis that did not respond to 60 mg per day of oral prednisone. Adding 0.6 mg of oral colchicine twice per day led quickly to the resolution of the condition. A chronic therapy of 5 mg per day of prednisone and oral colchicine, and 0.6 mg twice per day, was sufficient for healing [108]. A case series study showed that 12 out of the 13 patients treated reported a positive outcome to oral colchicine, with a dosage of 1.2 to 2.4 mg per day [109]. A randomized, prospective, controlled trial divided 41 patients into two groups and compared the efficacy of oral colchicine, 0.5 mg twice per day, and topical emollients. No difference in treatment outcome was observed among the groups. A complete response was achieved in five patients in the colchicine group and in seven in the control group. Three patients of the colchicine group relapsed after drug interruption, suggesting that the therapy was somehow effective [110]. The efficacy of colchicine in leukocytoclastic vasculitis remains controversial.

#### 4.12.3. Urticaria Vasculitis

Urticaria vasculitis (UV) is a subtype of cutaneous small-vessel vasculitis. It is characterized by erythematous patches, or weals, resembling urticaria but lasting more than 24 h, and after healing, it results in hyperpigmented macules. It can be classified as normo- or hypocomplementemic urticarial vasculitis [111].

Antihistamines or non-steroidal anti-inflammatory drugs may relieve symptoms. For the long-term control of severe forms, drugs such as dapsone, hydroxychloroquine, indometacin, and corticosteroids have been proposed [112].

Wiles and colleagues reported a case series study of two patients that were resistant to systemic steroid treatment that responded very quickly to oral colchicine [113].

Fujii and colleagues used colchicine in a case of hypocomplementemic urticarial vasculitis with Sjogren’s syndrome. Oral prednisolone (35 mg per day) improved the cutaneous eruptions; it was then quickly tapered by its combination with colchicine (1 mg per day) without recurrence of the symptoms [114]. A Japanese group reported using colchicine in a patient that did not respond to a 75 mg-per-day oral dapsone therapy, with optimal results [115]. Asherson et al. reported the the effectiveness of 0.5 mg of oral daily colchicine, also in a case of normo-complemented urticarial vasculitis that was resistant to indomethacin, steroids, dapsone, and hydroxychloroquine [116].

In a retrospective cohort study including 57 subjects suffering from hypocomplementemic urticarial vasculitis, the efficacy of colchicine was reported to be similar to that of corticosteroids [117].

#### 4.12.4. Behçet’s Disease

Behçet’s disease (BD) is a chronic-relapsing inflammatory disease of unknown etiology. BD is characterized by frequent episodes of oral and/or genital ulcers, associated with ocular, joint, skin, and vascular involvement [118]. It is considered a form of vasculitis which primarily targets small arteries, but it can affect both arteries and veins of all sizes [119]. In BD, colchicine is a first-line treatment, especially for the cutaneous symptoms. Numerous case reports and case series studies described the use of colchicine in this disease with good results [120,121]. Furthermore, clinical trials confirmed the efficacy of this drug [122,123,124]. Therefore, BD is one of the main indications for colchicine treatment.

#### 4.12.5. Pigmented Purpuric Dermatoses

Pigmented purpuric dermatoses (PPD) are a group of benign, chronic, purpuric skin eruptions characterized by red-to-purple macules, patches, and petechiae. Most cases are idiopathic, but they may be associated with coagulopathies or thrombocytopenia. Histologically, it is capillaritis with a secondary extravasation. Many subtypes are described (Schamberg disease, purpura annularis telangiectodes of Majocchi, lichen aureus, pigmented purpuric lichenoid dermatitis of Gougerot–Blum, eczematoid-like purpura of Doucas and Kapetanakis, disseminated pruriginous angiodermatitis, unilateral linear capillaritis, and granulomatous pigmented purpura). Given the benign and chronic nature of the condition, no gold-standard treatment is reported to treat PPD [125,126]. An Indian group described the case of a 45-year-old man affected by a granulomatous pigmented purpuric dermatosis that did not respond to topical betamethasone valerate 0.1% cream, along with compression stockings, that healed after 3 months of treatment with oral colchicine at 1 mg per day, as well as calcium dobesilate at 1 g per day [127].

#### 4.12.6. Other Vasculitis

An Italian group described the use of oral colchicine for non-cryoglobulinemic vasculitis in Sjögren’s syndrome [128]. A precise diagnosis was not made due to the patient’s refusal of a skin biopsy.

A Japanese group reported the efficacy of colchicine at 0.5 mg, once per day, in treating a 10-year-old boy with an IgA vasculitis refractory to factor XIII concentrate, with intravenous immunoglobulin G, and mycophenolate mofetil. [129]. Various other cases reported the successful treatment of IgA vasculitis with colchicine. An Israeli group reported the successful treatment of two teenage girls with a combination of 2 mg of colchicine and 100 mg per day of acetylsalicylic acid [130]. Pyne and colleagues reported good results in a 42-year-old patient that was administered 0.5 mg of colchicine per day [131]. Saulsbury reported two children with an optimal response to oral colchicine [132].

Hazen and Michel described a series study of six patients affected by necrotizing vasculitis who were treated with oral colchicine, with clinical improvements reported in more than 80% of them [133].

The Vasculitis Clinical Research Consortium aims to roll a multicenter, sequential, multiple assignment randomized trial with an enrichment design to compare the efficacy of three drugs (azathioprine, colchicine, and dapsone) in the management of isolated cutaneous small- or medium-vessel vasculitis, including cutaneous small-vessel vasculitis, immunoglobulin A (IgA) vasculitis (skin-limited Henoch–Schönlein purpura), and cutaneous polyarteritis nodosa, helping to identify the most effective drug to treat these kinds of conditions [134].

### 4.13. Erythema Induratum

Erythema induratum (EI), also called nodular vasculitis (NV), refers to a chronic lobular panniculitis that may or may not be associated with vasculitis. If it is secondary to tuberculosis, the disease is called Bazin’s EI; otherwise, it is called EI of Withfield [135]. A case series study described three patients with EI, one reporting a previous tubercular infection and two with no infection. Traditional therapies, including multidrug anti-tubercular therapies (rifampicin, isoniazid, ethambutol, and pyrazinamide), corticosteroids, methotrexate, cyclosporine, mycophenolate, and dapsone showed minor-to-no improvements. In contrast, the treatment with colchicine at 0.5 mg twice per day combined with a low dose of corticosteroids led to a complete/almost complete remission in all the patients [136]. A prospective study will be necessary to confirm the results obtained by the available reports.

### 4.14. Cutaneous Amyloidosis

Amyloidosis is a condition characterized by the stacking of various insoluble proteins (amyloids) in amounts that cause dysfunction in an organ. This stacking may involve all organs or may be confined to a single one, depending on the insoluble protein involved [137]. The two most common primary cutaneous amyloidoses are lichen and macular amyloidosis, reporting similar histological features [138]. A case series study described 15 patients with primary localized cutaneous amyloidosis, of which eight had macular amyloidosis and seven had lichen amyloidosis. They received oral colchicine at 0.5 mg twice per day for 3 months. Pruritus, and size of the papules, decreased in both subgroups of patients [139]. A prospective study will be necessary to confirm the results obtained by the current reports.

### 4.15. Cutaneous Sarcoidosis

Sarcoidosis is a condition characterized by the formations of granulomas in various parts of the body, including the skin (cutaneous sarcoidosis) [140]. Various treatments have been proposed, such as corticosteroids, immunosuppressants, and biologic drugs [141]. Wise reported three patients affected by facial cutaneous sarcoidosis that rapidly responded, with a long remission, to combined therapy with systemic colchicine and a topical corticosteroid ointment [142]. A prospective study will be necessary to confirm the results obtained by the current reports.

### 4.16. Hereditary Angioedema

Hereditary angioedema is a spectrum of genetic-based conditions characterized by the recurrent attacks of self-limiting oedemas of the skin, gastrointestinal tract, and airways. It is classified into three types based on the pathogenesis [143]. Although it is possible to use some drugs, such as an androgen, an antifibrinolytic, or a progestin for the prevention of acute episodes; in case of an acute attack, a plasma-derived C1-esterase inhibitor, a kallikrein inhibitor, or a BDKRB2 antagonist should be administered as soon as possible [144].

A Turkish group [145] reported using oral colchicine with a partial remission of symptoms in a patient suffering from type 1 hereditary angioedema concomitantly affected by familial Mediterranean fever, showing some usefulness of the drug in this condition.

### 4.17. Acquired Perforating Dermatosis

Perforating dermatoses (PD) are a heterogeneous group of cutaneous diseases defined by the transepidermal elimination of dermal tissue. Four classical forms are described: the acquired reactive perforating collagenosis that eliminates collagen fibers, elastosis perforans serpiginosa that eliminates elastic fibers, Kyrle disease that eliminates keratin, and perforating folliculitis [146]. No first-line treatment is currently available for PD [147]. Grover and colleagues reported the case of a 68-year-old woman affected by diabetes mellitus and chronic kidney disease that successively developed acquired reactive perforating collagenosis. The patient did not respond to oral antihistamines, potent topical corticosteroids, and topical retinoids, so a therapy of oral colchicine, initially 0.5 mg once a day, then twice a day, was administered, with a rapid and complete response in 4 weeks [148]. Gil and colleagues also reported the use of 1 mg of oral colchicine twice daily, associated with a potent topical steroid, for the management of an aspecific acquired perforating dermatosis, with its reasonable control after 2 months of the treatment [149]. Further studies are needed to confirm these results.

### 4.18. Linear IgA Bullous Disease

Linear IgA bullous disease (LIBD), or chronic bullous dermatosis in children, is an autoimmune condition characterized by blisters on the skin and mucous membranes. The diagnostic finding is the presence of the immunoglobulin A deposition at the dermo-epidermal junction using immunofluorescence. It may be associated with inflammatory bowel disease, solid and lymphoid malignancies, and rheumatoid arthritis. No association with gluten-sensitive enteropathy is reported [150,151]. The traditional therapy relies on oral dapsone; alternative treatments have been proposed in case of inefficacy, AEs, or contraindication (i.e., favism), among which colchicine is included. Banodkar and al-Suwaid reported a case series study of eight patients, five boys and three girls, aged between 3 and 9 years, treated with 0.5 mg of oral colchicine twice per day. Five patients responded to the monotherapy, while three patients needed a small dose of steroids to reach remission. No severe side effects were observed [152]. Aram reported a 25-year-old woman suffering from LIBD that was treated with dapsone and developed a hemolytic reaction. Dapsone was suspended, and a treatment of colchicine 0.5 mg three times per day was administered, leading to a quick resolution of cutaneous symptoms. Treatment was then discontinued, and the patient relapsed; a further cycle of oral colchicine led to the complete resolution of LIBD [153]. Zeharia and colleagues treated a 3.5--year-old boy with colchicine, 0.5 mg twice per day, with good results [154]. Ang and Tay reported the successful treatment of oral colchicine, 0.5 mg twice per day, in a 5-year-old girl as a second-line treatment after developing dapsone-related anemia [155]. Colchicine is a safe treatment that may be proposed as a second-line treatment when patients fail first-line treatments, develop side effects from the treatment, or suffer from favism.

### 4.19. Pemphigus

**Figure 5:**
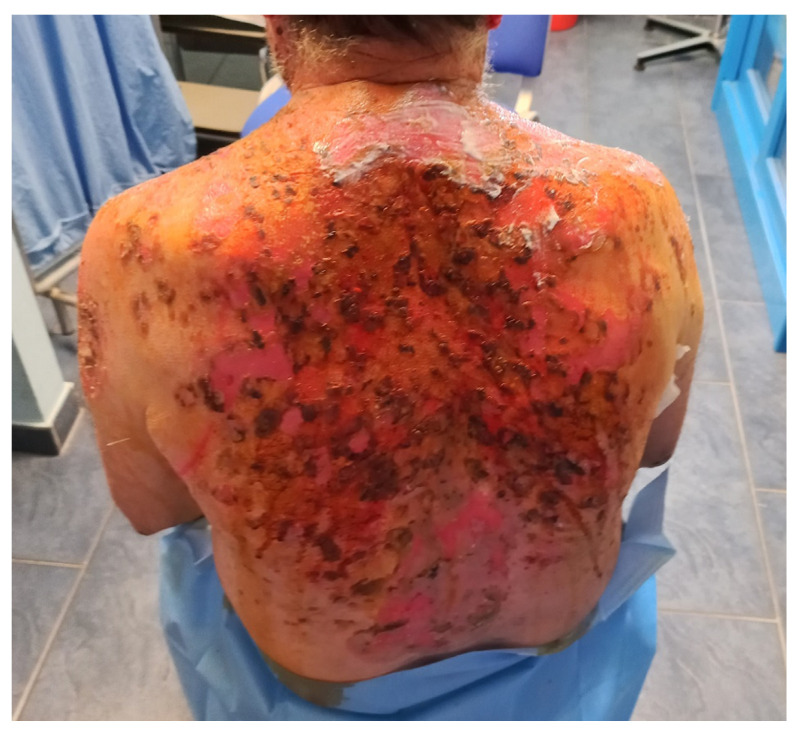
Pemphigus (courtesy of Dr. Bennardo).

Pemphigus is a group of autoimmune diseases characterized by the formation of erosions and/or flaccid bullae of the skin and/or mucosae (Figure 5). All variants of pemphigus are characterized by the development of autoantibodies to the desmosomal proteins of the epidermis [156]. An Israeli group reported treating two cases of IgA pemphigus with 0.5 mg of colchicine three times per day, with the resolution of the condition in two weeks. [157]. A Japanese group used colchicine and a topical steroid in the treatment of pemphigus foliaceous, with good results [158].

### 4.20. Bullous Pemphigoid

Bullous pemphigoid (BP) is an autoimmune disease characterized by subepidermal blistering. BP is caused by autoantibodies that are directed to the antigens of the hemidesmosome, BP180 and BP230 [159]. A Greek group reported a combination therapy of oral steroids and an immunosuppressant in 15 patients to treat mucosal membrane pemphigoid. Out of the various drugs used, colchicine resulted in the best control rate of the condition (8 out of 12, 66%) [160]. A prospective study will be necessary to confirm the results obtained by the current reports.

### 4.21. Epidermolisis Bullosa

Evidence for treating epidermolysis bullosa (EB), both congenital and acquisita, with colchicine is limited to case series studies. However, they suggest that the drug may be an effective treatment modality in EB [161]. A Japanese group described a 65-year-old woman with EB acquisita that did not respond to steroids; the treatment with 1 mg per day of colchicine led to good results [162]. Megahed and Scharffetter-Kochanek reported the cases of a 71-year-old-woman and a 65-year-old-man, who were both resistant to traditional therapies, that responded to a dosage of 2 mg of colchicine per day, lowered down to 1 mg per day after six months [163]. Cunningham et al. reported four cases of EB acquisita that were successfully treated with variable doses of oral colchicine (from 0.5 to 1.5 mg per day) as a single treatment or associated as with other drugs, such as cyclophosphamide, dapsone, and prednisone [164]. Kaneko et al. treated two patients with recessive dystrophic EB that were also affected by amyloid nephropathy with 1.5 mg of colchicine per day, and two patients affected by the same condition that did not take the drug, showing that colchicine could also act on the nephropathy [165]. Arora and colleagues reported a 42-year-old woman affected by EB acquisita that responded to 0.5 mg of oral colchicine three times per day [166], while Adachi et al. reported the case of a 58-year-old male who responded to 1 mg per day [167]. Kim treated a 42-year-old woman suffering from junctional EB with oral colchicine at 0.5 mg, reaching a dramatic response [168]. Literature reports suggest that colchicine may help different types of EB, such as junctional EB or recessive dystrophic EB.

### 4.22. Dermatitis Herpetiformis

Dermatitis herpetiformis (DH) is a vesico-bullous disease mainly associated with celiachia. Only one case series of DH with four patients is reported; they were treated with 0.6 mg of colchicine three or four times per day, leading to improvement in three out of four patients. All patients reported diarrhea that led to the dose reduction/suspension of the drug, with a recurrence of the condition [169]. A prospective study will be necessary to confirm the results obtained by this case series study.

### 4.23. Subcorneal Pustular Dermatosis

**Figure 6:**
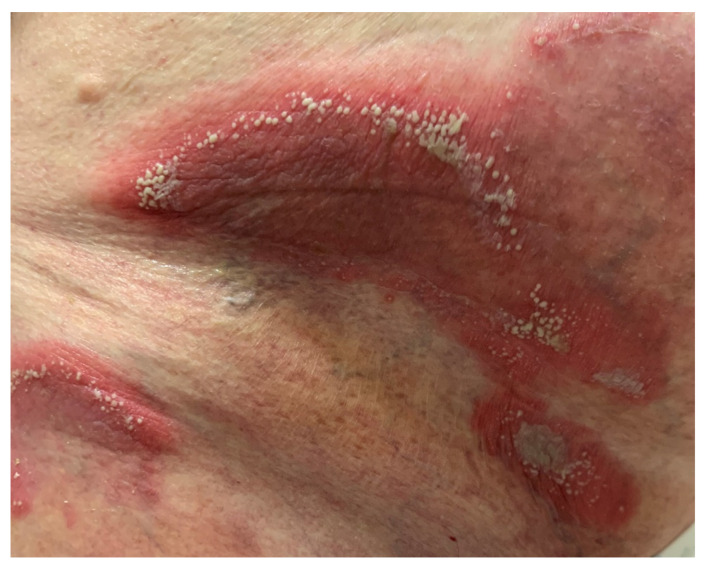
Subcorneal Pustular Dermatosis (courtesy of Dr. Dastoli).

Subcorneal pustular dermatosis, or Sneddon–Wilkinson’s disease, is a condition characterized by pustular lesions on the trunk and flexural areas, such as the armpits and groin (Figure 6) [170]. Pavithran reported a patient developing AEs with a combination therapy of dapsone and corticosteroids. Oral colchicine at 0.5 mg twice per day was started, with a complete resolution. The dose was dropped to 0.5 mg once per day for maintenance, with no recurrence [171]. Further studies are necessary to confirm the results obtained by this case report.

### 4.24. Stomatitis

Colchicine is currently used for different types of mouth inflammatory conditions, with good results. Quintana-Ortega and colleagues performed a multicenter study on 13 young patients affected by periodic fever, aphthous stomatitis, pharyngitis, and cervical adenitis (PFAPA) syndrome. The mean age at the start of the colchicine treatment was 6, and the median dosage was 0.02 mg per kg per day. Treatment was continued for 1 year; as a result, a decreased number of flares and a shortened duration of the disease episodes were recorded [172]. This efficacy was confirmed by an extensive study involving 400 children affected by PFAPA. The patients started on colchicine treatment for 12 consecutive months (0.5 mg per day of colchicine in children < 5years of age, 1 mg per day for children between 5 and 10 years of age, and 1.5 mg per day for children > 10 years of age). Only 358 patients continued the prophylactic therapy due to AEs, reducing the mean time of recurrence from 18.8 ± 7.9 to 49.5 ± 17.6 days. These results were particularly significant in the Mediterranean fever (MEFV) variant [173]. Different other studies confirmed these results [174,175,176]. Recurrent aphthous stomatitis (RAS) is a common disorder of the oral mucosa characterized by recurrent painful superficial necrotizing ulcerations with a red halo. RAS has been treated with colchicine. Forty-eight patients with RAS were divided into two groups. One group took oral colchicine at 0.5 mg, three times per day; the other group took a placebo. Colchicine had a therapeutic effect on aphthae, and the recovery period was shortened [177]. Pakfetrat et al. performed a double-blind, randomized trial in which 34 patients were randomly divided into two groups for the treatment with prednisolone or colchicine for three months. Both drugs significantly reduced the symptoms. The rate of AEs was higher in colchicine (52.9% vs. 11.8%), suggesting that low-dose prednisolone should be used as a first treatment [178]. Ruah et al. reported three patients with RAS treated with colchicine, obtaining optimal clinical results. One patient discontinued therapy due to AEs, while the other two remained disease-free at 3 and 5 years [179]. Katz and colleagues reported an open crossover trial in which 20 RAS patients took oral colchicine at 0.5 mg three times per day, with 95% of patients reporting an improvement in pain, and 90% of patients reporting an improvement in the aphthae count; only 20% of patients experienced mild AEs but this did not lead to treatment discontinuation [180].

**Table 2:** Levels of evidence and grades of recommendation of the various skin conditions.

Condition	References	Level of Evidence	Grade of Recommendation
**Psoriasis**	[21,22,23,24,25,26,27,28,29,30]	4	D
**Palmoplantar Pustulosis**	[33,34,35,36,37,38]	3b	D
**Neutrophilic Eccrine Hidradenitis**	[39]	4	C
**Plantar Eccrine Hidradenitis**	[40]	4	C
**Hidradenitis Suppurativa**	[50,51,52]	2b	D
**Acne**	[59,60,61]	2b	D
**Urticaria**	[64,65,66,67]	3b	D
**Pyoderma Gangrenosum**	[70,71,72,73]	4	C
**Sweet Syndrome**	[76,77,78,79,80]	3b	C
**Erythema Nodosum**	[83]	4	D
**Actinic Keratosis (topical)**	[90,91,92,93]	1b	B
**Vasculitis**	[103,104,108,109,110,113,114,115,116,117,120,121,122,123,124,127,128,129,130,131,132,133]	3b	C
**Erythema Induratum**	[136]	4	C
**Cutaneous Amyloidosis**	[139]	4	C
**Cutaneous Sarcoidosis**	[142]	4	C
**Hereditary Angioedema**	[145]	4	C
**Acquired Perforating Dermatosis**	[148,149]	4	C
**Linear IgA Bullous Disease**	[152,153,154,155]	4	C
**Pemphigus**	[157,158]	4	C
**Pemphigoid**	[160]	4	C
**Epidermolysis Bullosa**	[162,163,164,165,166,167,168]	4	C
**Dermatite Herpetiformis**	[169]	4	C
**Subcorneal Pustular Dermatosis**	[171]	4	C
**Stomatitis**	[172,173,174,175,176,177,178,179,180]	1b	B

## 5. Conclusions

Oral colchicine may be considered a safe second- or third-line treatment in several skin diseases, including eccrine hidradenitis, PG, EN, EI, storage diseases, perforating dermatoses, bullous diseases, and pustular dermatoses. Although mainly based on small case series studies, the evidence seems to be promising. For other conditions, such as psoriasis, HS, vasculitis, palmoplantar pustulosis, acne, and urticaria, contrasting results exist. For psoriasis, pustular variants (as reported for other pustular dermatoses) seem to respond better to colchicine, while the efficacy of this drug in the management of arthropathic psoriasis has not been fully proven. Oral colchicine is usually used with dosages between 0.5 mg and 2.5 mg per day, with dosages higher than 2 mg mainly used to manage patients affected by flares of familial Mediterranean fever. It would be wise to not overcome the dose of 2 mg per day due to the risk of side effects. In most dermatological patients, a dosage of 1–1.5 mg per day was usually enough to reach clinical efficacy. If colchicine must be used for longer period of time, it is better to lower the daily dose to 0.5–1 mg per day, in order to reduce the risk of systemic side effects. Topical colchicine might be an interesting, safe, and effective second-line treatment for psoriatic plaques, although it has rarely been investigated. While not responding to monotherapy, HS seems to respond when colchicine is associated with antibiotics. Vasculitis seems to have a different response profile according to the subtype, with conditions such as Behçet’s disease, where colchicine is the main treatment; urticarial vasculitis, in which colchicine is considered an effective treatment; or leukocytoclastic vasculitis, where the efficacy of the drug remains controversial. An upcoming randomized trial of the Vasculitis Clinical Research Consortium should shed light on the usefulness of this molecule. Colchicine may be considered as first-line therapy in other conditions, such as aphthous stomatitis, both in the syndromic variants and in VAS. Topical colchicine may be considered as an effective alternative to treat actinic keratosis. The rate of side effects reported after using oral colchicine may be considered low and, in most cases, it is limited to gastrointestinal manifestations, especially if the dosage does not exceed 1.5 mg per day. Further trials are, however, necessary in order to confirm the usefulness of this drug in various skin diseases, especially for those where current treatment alternatives are lacking, or they do not provide the patients with good, sustained results.

## Figures and Tables

**Table 1:** Level of evidence for rating studies and grading recommendations.

Level	Type of Evidence
**1a**	Systematic review with homogeneity of randomized controlled trials
**1b**	Individual randomized control trial with a narrow confidence interval
**1c**	All-or-none related outcome
**2a**	Systematic review with homogeneity of cohort studies
**2b**	Individual cohort studies and low-quality randomized clinical trials
**2c**	Ecological studies
**3a**	Systematic review of case-control or retrospective studies
**3b**	Individual case-control or retrospective studies
**4**	Case series and case reports
**5**	Expert opinion
**Grades of Recommendation**	
**A**	Consistent level 1 studies
**B**	Consistent level 2 or 3 studies or extrapolation from level 1 studies
**C**	Level 4 studies or extrapolation from level 2 or 3 studies
**D**	Level 5 evidence or contrasting results reported among studies
